# Effects of in vitro endochondral priming and pre-vascularisation of human MSC cellular aggregates in vivo

**DOI:** 10.1186/s13287-015-0210-2

**Published:** 2015-11-05

**Authors:** Fiona E. Freeman, Ashley B. Allen, Hazel Y. Stevens, Robert E. Guldberg, Laoise M. McNamara

**Affiliations:** Centre for Biomechanics Research (BMEC), Biomedical Engineering, College of Engineering and Informatics, National University of Ireland Galway, Galway, Ireland; Wallace H. Coulter Department of Biomedical Engineering, Parker H. Petit Institute for Bioengineering & Bioscience, Georgia Institute of Technology, 315 Ferst Drive NW, Atlanta, GA 30332 USA; George W. Woodruff School of Mechanical Engineering, Parker H. Petit Institute for Bioengineering & Bioscience, Georgia Institute of Technology, 315 Ferst Drive NW, Atlanta, GA 30332 USA

**Keywords:** Tissue engineering, Endochondral ossification, Endothelial cells, Mesenchymal cells, Vasculogenesis, Osteogenesis, Cell viability

## Abstract

**Introduction:**

During endochondral ossification, both the production of a cartilage template and the subsequent vascularisation of that template are essential precursors to bone tissue formation. Recent studies have found the application of both chondrogenic and vascular priming of mesenchymal stem cells (MSCs) enhanced the mineralisation potential of MSCs in vitro whilst also allowing for immature vessel formation. However, the in vivo viability, vascularisation and mineralisation potential of MSC aggregates that have been pre-conditioned in vitro by a combination of chondrogenic and vascular priming, has yet to be established. In this study, we test the hypothesis that a tissue regeneration approach that incorporates both chondrogenic priming of MSCs, to first form a cartilage template, and subsequent pre-vascularisation of the cartilage constructs, by co-culture with human umbilical vein endothelial cells (HUVECs) in vitro, will improve vessel infiltration and thus mineral formation once implanted in vivo.

**Methods:**

Human MSCs were chondrogenically primed for 21 days, after which they were co-cultured with MSCs and HUVECs and cultured in endothelial growth medium for another 21 days. These aggregates were then implanted subcutaneously in nude rats for 4 weeks. We used a combination of bioluminescent imaging, microcomputed tomography, histology (Masson’s trichrome and Alizarin Red) and immunohistochemistry (CD31, CD146, and α-smooth actin) to assess the vascularisation and mineralisation potential of these MSC aggregates in vivo.

**Results:**

Pre-vascularised cartilaginous aggregates were found to have mature endogenous vessels (indicated by α-smooth muscle actin walls and erythrocytes) after 4 weeks subcutaneous implantation, and also viable human MSCs (detected by bioluminescent imaging) 21 days after subcutaneous implantation. In contrast, aggregates that were not pre-vascularised had no vessels within the aggregate interior and human MSCs did not remain viable beyond 14 days. Interestingly, the pre-vascularised cartilaginous aggregates were also the only group to have mineralised nodules within the cellular aggregates, whereas mineralisation occurred in the alginate surrounding the aggregates for all other groups.

**Conclusions:**

Taken together these results indicate that a combined chondrogenic priming and pre-vascularisation approach for in vitro culture of MSC aggregates shows enhanced vessel formation and increased mineralisation within the cellular aggregate when implanted subcutaneously in vivo.

## Introduction

Tissue engineering and regenerative medicine have significant potential to treat bone pathologies by exploiting the capacity for bone progenitors to grow and produce tissue constituents under specific biochemical and physical conditions [1–19]. However, the regenerated bone tissue produced through such approaches is limited, due to the degradation occurring in the centre of the constructs and loss of cell viability due to hypoxia occurring within the constructs, which arise from lack of vascularisation [13, 20–26] and a lack of mechanical integrity of the regenerated tissue. As a result such strategies are not yet widely used for clinical treatment of large bone defects.

Endochondral ossification is the process by which all long bones are formed during early fetal development. It relies on the production of a cartilage template, which is followed by vessel invasion. This occurs once the cartilage template has formed; endothelial cells invade through the cartilage canals already present in the developing bone tissue [27–30], and this process typically occurs between 14 and 18 days of embryogenesis [30, 31]. Both cartilage template formation and vascularisation must occur before bone tissue can be formed. Recent findings have suggested that mimicking the cartilage template formation phase of the endochondral ossification process, by chondrogenically priming mesenchymal stem cells (MSCs), may be an effective approach to overcome issues such as poor oxygen and nutrient supply in bone tissue-engineered constructs [26, 32–34] as chondrocytes are physiologically functional even at reduced oxygen tension [35]. However, even with chondrogenic priming, construct degradation and an uneven distribution of bone mineral have been reported throughout the construct after implantation [25, 26, 33]. In a recent study we found that chondrogenic priming of BALBc mice MSCs and human MSCs in vitro for specific durations (14 and 21 days) can influence their mineralisation capacity and produce a construct that is mineralised throughout the core to a greater degree than culturing the cells in osteogenic growth factors alone [34]. In vivo studies demonstrated that chondrogenically primed constructs seeded with embryonic stem cells [36], chick embryonic stem cells [37] and human MSCs [25, 26, 33, 38] subsequently mineralised and in some cases formed bone marrow cavities [37, 38] following subcutaneous implantation in rodent animal models. Chondrogenically primed rat MSCs cultured on a PLGA scaffold were found to have increased bone healing in both a 5-mm and 15-mm rat femur defect [39]. Similar rapid healing was also reported when chondrogenically primed human MSC cellular aggregates were implanted in a 6-mm rat femur defect [40].

Without a suitable vascular supply, cells within tissue engineered constructs lack the necessary requirements to regenerate bone tissue and readily perish when implanted in vivo [41, 42]. In vitro co-culture studies have investigated whether pre-vascularising three-dimensional tissue-engineered constructs, such as trabecular bone [43], polycaprolactone (PCL) [44], poly(LLA-co-DXO) [45], collagen glycosaminoglycan (GAG) [46, 47], and hydroxyapatite [48] scaffolds in vitro, through the co-culture of MSCs and human umbilical vein endothelial cells (HUVECs), would allow faster host integration post-implantation [43–48]. It has been shown that pre-vascular networks can be formed in a subcutaneous animal model in vivo when human MSCs [43, 45, 46, 48] are first co-cultured with HUVECs in vitro. Moreover, in vitro co-culture studies of HUVECs and MSCs have detected an upregulation of the early osteogenic growth factor alkaline phosphatase (ALP) in both two- and three-dimensional culture [49–53].

While current bone regeneration strategies have sought to incorporate either the production of the cartilage template or the vascularisation of the construct, no strategy has sought to incorporate both events simultaneously, even though both are crucial precursors for bone formation during endochondral ossification in vivo. In a recent in vitro study, we found that chondrogenic priming (for 21 days) together with co-culture of human MSCs and HUVECs significantly increased the osteogenic potential of the culture compared to chondrogenic priming alone [54]. This study also reported that both MSCs and HUVECs must be added to the formed cartilage template for the formation of rudimentary vessels to occur in vitro. We found that the application of both chondrogenic and vascular priming of MSCs enhanced the mineralisation potential of MSCs in vitro whilst also allowing for immature vessel formation. However, the in vivo viability, vascularisation and mineralisation potential of MSC aggregates that have been pre-conditioned in vitro by a combination of chondrogenic and vascular priming has yet to be established.

In this study, we test the hypothesis that a tissue regeneration approach that incorporates both chondrogenic priming of MSC aggregates, to first form a cartilage template, and subsequent pre-vascularisation of the cartilage constructs, through the co-culture of HUVECs in vitro, will improve cell survival, vessel infiltration and thus mineral formation once implanted in vivo. The specific objectives of this study are to assess these outcome measures within a subcutaneous implantation nude rat model.

## Methods

### Cell culture

#### Human donor MSCs

Bone marrow-derived human MSCs harvested from two male donors, 20–25 years old, with established multi-potency, were commercially available and purchased from the Texas A&M University Health Science Centre (Temple, TX, USA). As the human MSCs were bought from Texas A&M University Health Science Centre, all ethical approval was conducted by them. The human MSCs were expanded in minimum essential medium alpha (αMEM; Invitrogen, Carlsbad, CA, USA) containing 16.7 % fetal bovine serum (FBS; Atlanta Biologicals, Lawrenceville, GA, USA) and 100 units/mL penicillin/100 μg/mL streptomycin/2 mM L-glutamine (PSL; Invitrogen) at 37 °C and 5 % CO_2_. For all cell culture performed in this study, cell culture medium was changed twice weekly unless stated otherwise. At passage 2, cells from each donor were detached using 0.25 % trypsin- ethylenediaminetetraacetic acid (EDTA; Invitrogen) and combined 1:1 to produce a pooled human MSC population. MSCs were further cultured to passage 3–4.

#### Cell labelling

Human MSCs were co-transduced using lentiviral vector containing green fluorescent protein (GFP) and firefly luciferase (Luc) downstream of the ubiquitin promoter as previously described [55–57]. Briefly, human MSCs were suspended in polybrene and a viral vector at a multiplicity of infection (MOI) of 20 and incubated in flasks at a density of 10,000 cells/cm^2^ overnight (Sigma, St. Louis, MO, USA). Medium was changed daily for 3 days, after which the labelling efficiency of GFP/Luc was determined using fluorescent microscopy. GFP/Luc-labelled human MSCs were replated at a seeding density of 500 cells/cm^2^ and were further cultured to passage 3–4. These GFP/Luc-labelled human MSCs were only used in the constructs tested for bioluminescent imaging (BLI).

#### HUVEC culture

HUVECs were commercially available and purchased from Lonza (Maryland, USA) and cultured in Clonetics endothelial growth medium (EGM) SingleQuotes (Lonza). As the HUVECs were purchased from Lonza, all necessary ethical approval was conducted by them. Media was replaced every 3 days and, upon reaching 80–90 % confluency, cells were passaged using 0.25 % trypsin-EDTA (Invitrogen). HUVECs were further cultured to passage 3.

### Aggregate formation

Once the human MSCs (labelled and unlabelled) reached a confluency of ~80 % the cells were trypsinised, counted, and centrifuged at 650 g at a temperature of 22 °C for 5 min. The cells were then resuspended in expansion media at a density of 0.25 × 10^6^ cells/mL. This cell suspension was divided into 1.5 mL tubes so that there were 250,000 cells in each tube, and these were then centrifuged for 5 mins (Eppendorf Centrifuge 5430R; Vashaw Scientific, Norcross, GA, USA) at 400 g to create cell aggregates. The media was removed from the tube carefully, so as to avoid the newly formed aggregate, and 0.5 mL of chondrogenic media was added. Chondrogenic medium consisted of a chemically defined medium, which contained high-glucose Dulbecco’s modified Eagle's medium (DMEM) GlutaMAXTM (Invitrogen), 10 ng/mL transforming growth factor (TGF)-β3 (Invitrogen), 50 μg/mL ascorbic acid (Sigma Aldrich), 4.7 μg/mL linoleic acid-oleic acid (Sigma Aldrich), 100 nM dexamethasone (Sigma Aldrich) and 1× insulin–transferrin–selenium (ITS; Invitrogen). For all experiments aggregate cultures were fed twice per week by performing a 50 % medium exchange. During each feed the aggregates were agitated, so as to prevent them from adhering to the micro-tube. This was achieved through aspirating the media beneath the aggregate with a micro-pipette.

**Fig. 1:**
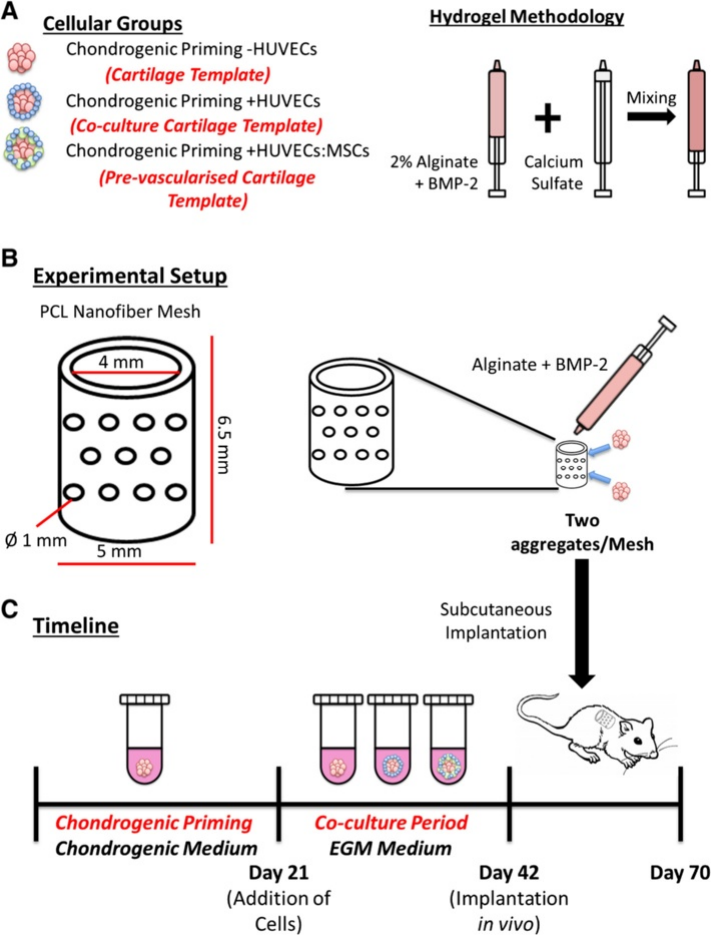
Schematic of the **a** cellular groups and hydrogel methodology, **b** experimental setup and **c** timeline of the experiment. *BMP* Bone morphogenetic protein, *EGM* Endothelial growth medium, *HUVEC* Human umbilical vein endothelial cell, *MSC* Mesenchymal stem cell, *PCL* Polycaprolactone

After 21 days, the aggregates were separated into three different experimental conditions: 1) CP21 – HUVECs (aggregates were chondrogenically primed for a period of 21 days and then cultured in EGM media for a further 21 days; hereafter known as the Cartilage Template group); 2) CP21 + HUVECs (aggregates were chondrogenically primed for 21 days after which 250,000 suspended HUVECs in EGM were added to the cellular aggregate and cultured in EGM for further 21 days; hereafter known as the Co-Culture Cartilage Template group); and 3) CP21 + HUVECs:MSCs (aggregates were chondrogenically primed for 21 days after which 250,000 suspended HUVECs and MSCs at a ratio of 1:1 (125,000:125,000 cells) in EGM were added and further cultured in EGM for 21 days; hereafter known as the Pre-vascularised Cartilage Template group); see Fig. 1.

For the co-culture groups, confluent layers of HUVECs/MSCs were trypsinised and counted. Cells were suspended depending on experimental conditions so that there were 0.5 × 10^6^ cells/mL. In the case of the CP21 + HUVECs:MSCs, the ratio of cells was 1:1 HUVECs:MSCs. Both the HUVECs and the MSCs added were at passage 3, which was the same passage of the MSCs used to form the original cellular aggregate. The cells were suspended in EGM media containing osteogenic growth factors and 20 % methocel, from a stock solution that was generated by dissolving 6 g carboxymethylcellulose (Sigma Aldrich) in 500 mL DMEM as previously described [58]. The addition of the methocel to the media increases the viscosity of the media and promotes the attachment of the cells to the already formed aggregate. After 24 h the medium that contained methocel was removed and was replaced with EGM media alone and this EGM media alone was used for the further 20 days of culture.

### Construct preparation

After 42 days of in vitro culture, the primed aggregates were prepared for implantation. A dual syringe approach, previously described by Kolambkar et al. [59], was adapted to imbed the cellular aggregates within hydrogels. Briefly, functionalised alginate (FMC Biopolymer; Sandvik, Norway) containing bone morphogenetic protein (BMP)-2 (Pfizer, MA, USA) at a concentration of 1.6 μg/100 μL was cross-linked by adding calcium sulphate (Sigma) to a final concentration of 8.4 mg/mL. Constructs were prepared by injecting 100 μL of cross-linked alginate into an electrospun, PCL nanofibre mesh tube [59], and two cellular aggregates from each group were placed within each alginate/mesh construct (Fig. 1). One group, which contained no aggregates within the mesh, was used as an acellular group (known as the Alginate group). These constructs were then incubated in culture medium within a 24-well ultralow-attachment plate (Corning, Lowell, MA, USA) for 2–6 h prior to implantation.

### Surgical procedures

All animal procedures were ethically approved and conducted in accordance with the Georgia Institute of Technology Institutional Animal Care and Use Committee protocol (#A13023). Ten 11-week-old female, athymic nude rats (Charles River Labs, Wilmington, MA, USA) were anaesthetised using isoflurane. Two incisions were made in the skin slightly lateral to the spine of each animal and a custom made tunnelling device was used to create four subcutaneous pockets. One construct (from each of the four groups) was placed in each pocket. Constructs were implanted in a balanced manner, such that each group contained an implant placed at each of the subcutaneous locations and samples were randomly distributed across the operated animals. Once the four constructs were implanted, incisions were closed using suture and wound clips.

### Bioluminescent imaging

Two rats received constructs with aggregates formed from GFP/Luc-labelled human MSCs (as discussed above) and were maintained under anaesthesia to perform day 0 BLI.

BLI was performed on the animals on days 0, 7, 14, and 21, following a previously developed approach [57]. Briefly rats were anaesthetised using isoflurane and 300 μL luciferin was injected subcutaneously in close proximity to the construct site. After 30 min, animals were positioned with their lateral side facing up and scanned using an IVIS Lumina machine (Caliper Life Sciences, Hopkinton, MA, USA). The animals were then repositioned so that their other side could be scanned. BLI images were evaluated by demarcation of a 4 cm^2^ elliptical region of interest (ROI) centred on each construct using Living Image software version 3.2 (Caliper Life Sciences). BLI counts were normalised by exposure time and ROI for each sample.

### Micro-computed tomography imaging

At 4 weeks post-surgery, eight rats (each rat contained the four groups) underwent a vascular perfusion protocol modified from that developed by Duvall et al. [60] and Allen et al. [57]. Briefly, the rats were put under anaesthesia and maintained at 4 % isoflurane. Once anaesthetised, the thoracic cavity was opened to insert an 18 gauge catheter (SURFLO Teflon IV catheter; Terumuo Medical, Somerset, NJ, USA) through the left ventricle of the heart into the ascending aorta. The inferior cava was cut and 0.9 % saline was perfused through the vasculature using a peristaltic pump (Masterflex, Cole Parmer, Vernon Hills, IL, USA) until the vasculature system was completely flushed clear. A solution of 0.9 % saline containing 0.4 % (w/v) papaverin hydrochloride was then perfused followed by 10 % neutral buffered formalin (NBF) for 5 min. Animals received a final perfusion of 20–25 mL radiopaque contrast agent Microfil (Flow Tech, Carver, MA, USA) and were left at 4 °C overnight. In this way, animals were euthanised by the combined effects of isoflurane overdose and exsanguination. Explants were extracted and incubated in NBF for 24 h before being imaged via micro-computed tomography (μCT) scans on a MicroCT42 (Scanco Medical, Brüttisellen, Switzerland) at 55 kVp, 145 μA, and a 12 μm voxel size. The volume of interest was defined as the construct and the minimal tissue surrounding the construct. Microfil has the same threshold as bone mineral and therefore to segment perfused vasculature from mineralised tissue within each construct two scans were analysed: calcified construct versus decalcified construct. The calcified constructs were scanned and post-processed using a threshold value that accurately depicted both the mineral content and the vessel volume by visual inspection of the two-dimensional greyscale tomograms (Scanco Medical MicroCT42). Noise was removed using a low-pass Gaussian filter (sigma = 1.2, support = 2). Next samples were decalcified in Immunocal (Formic acid bone Decalcifier, Decal Chemical Corporation) for 1 week with the decalcification solution replaced every day (decalcified constructs). After 1 week these decalcified constructs were scanned using the same settings, and post-processed at the same threshold as the calcified constructs to determine mineral content. Mineralised tissue content was determined by subtracting the bone volume of the decalcified scans from the calcified scans. Next the decalcified scans were post-processed at a threshold value that accurately depicted just the vessel volume upon visual inspection of the two-dimensional greyscale tomograms.

### Histochemical analysis

Following μCT scanning the samples were dehydrated and embedded in paraffin using an automatic tissue processor (Excelsior ES tissue processor, Thermo Scientific, Austin, TX, USA). All samples were sectioned with a thickness of 8 μm using a rotary microtome (Leica Microtome RM2235, Leica). Sections were stained with Masson’s Trichrome and Alizarin Red (all Sigma Aldrich).

### Immunohistochemical analysis

Immunohistochemical analysis was used to detect CD31, CD146 and α-smooth muscle actin. Sections were deparaffinised overnight before a series of rehydration steps through varying ethanol grades (100–50 %). The samples were then treated with 40 μg/mL proteinase K for 20 min at 37 °C (Sigma Aldrich), rinsed with phosphate-buffered saline (PBS)-Tween and blocked with PBS with 1 % w/v bovine serum albumin (BSA) and 3 % w/v normal goat serum (NGS; Sigma Aldrich) for 60 min. Sections were then incubated overnight at 4 °C with either rabbit polyclonal anti-CD31 (ab28364 Abcam, 1:50) or rabbit monoclonal anti-CD146 (ab75769 Abcam, 1:250). After three washing steps with PBS containing 1 % w/v BSA the sections were incubated with Dylight488 goat anti-rabbit secondary antibody (Jackson Immunoresearch, 115-485-209, 1/200), for 1 hour at room temperature in the dark. The samples were washed three times in PBS with 1 % w/v BSA, and the slides were then incubated overnight at 4 °C with mouse monoclonal anti-α-smooth muscle actin antibody (ab7817 Abcam, 1:50). After three washing steps with PBS with 1 % w/v BSA the sections were incubated with Dylight549 goat anti-mouse secondary antibody (Jackson Immunoresearch, 115-505-062, 1/200), for 1 hour at room temperature in the dark. Finally samples were washed three times with PBS with 1 % w/v BSA and the sections were mounted using 4′,6-diamidino-2-phenylindole (DAPI) mounting media (Sigma Aldrich).

### Statistical analysis

Results are expressed as mean ± standard error. All μCT quantitative analyses were examined using one-way analyses of variance (ANOVA) with the addition of Tukey’s correction for multiple comparisons testing. BLI quantitative analysis was examined using two-way ANOVA with the addition of Tukey’s correction for multiple comparisons testing. All analyses were performed using GraphPad (GraphPad Software, La Jolla California USA, www.graphpad.com). For all comparisons, the level of significance was *p* ≤ 0.05.

## Results

### Construct morphology

#### Prior to implantation

**Fig. 2:**
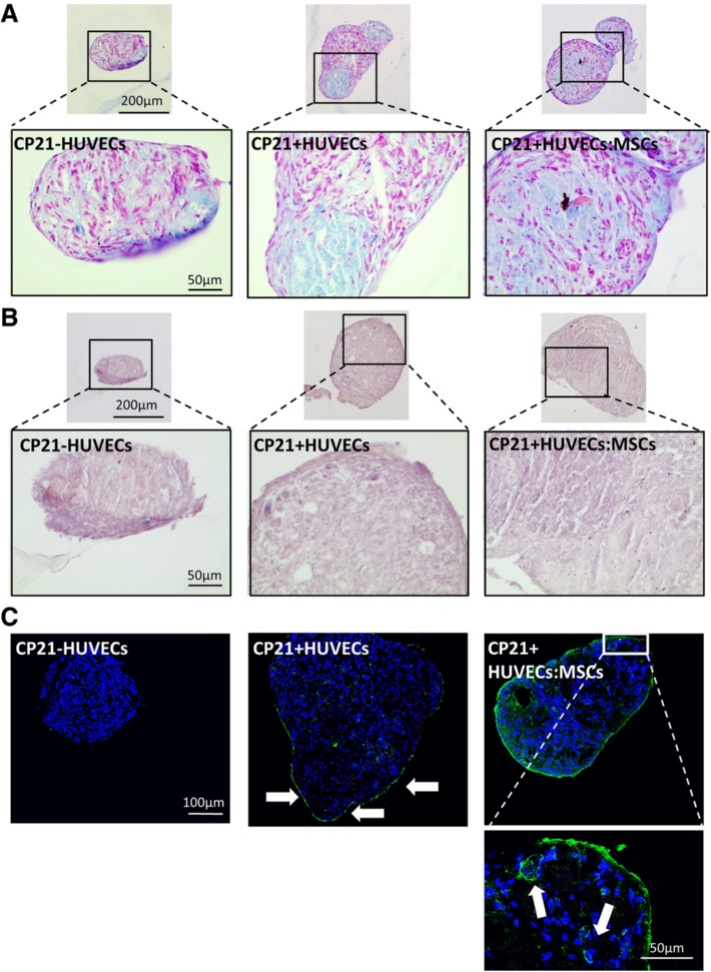
**a** Alcian Blue and **b** Alizarin Red staining of all three groups on the day of implantation (following 3 weeks of in vitro priming). Images were taken at a magnification of 10× and 40×. **c** Representative images of CD31+ (*green*) stained sections in the CP21 + HUVECs:MSCs group prior to implantation. Arrows denote the presence of positive CD31+ staining. Each section is 8 μm thick and each of the images was taken at a magnification of 20× and 60×. Nuclear counterstain: DAPI (*blue*). *CP21* Chondrogenically primedat day 21, *HUVEC* Human umbilical vein endothelial cell, *MSC* Mesenchymal stem cell

All three experimental groups stained positive blue for sGAG and Alizarin Red prior to implantation after 42 days of culture (Fig. 2a,b). There was no significant difference in sGAG or calcium production after the 6 weeks of culture.

There was no positive (green) staining for CD31+ in the CP21 – HUVECs group cultured as there were no endothelial cells present (Fig. 2c). However, for both the CP21 + HUVECs and the CP21 + HUVECs:MSCs groups there was positive (green) staining seen around the periphery (indicated by arrows in Fig. 2c) and, after 3 weeks of co-culture, both groups had positive green staining present within the centre of the aggregates. However, the CP21 + HUVECs:MSCs group was the only group to have structures characterised by a circular CD31+ positive wall with irregularly shaped nuclei present within the lumen (indicated by arrows in Fig. 2c).

#### Post implantation

**Fig. 3:**
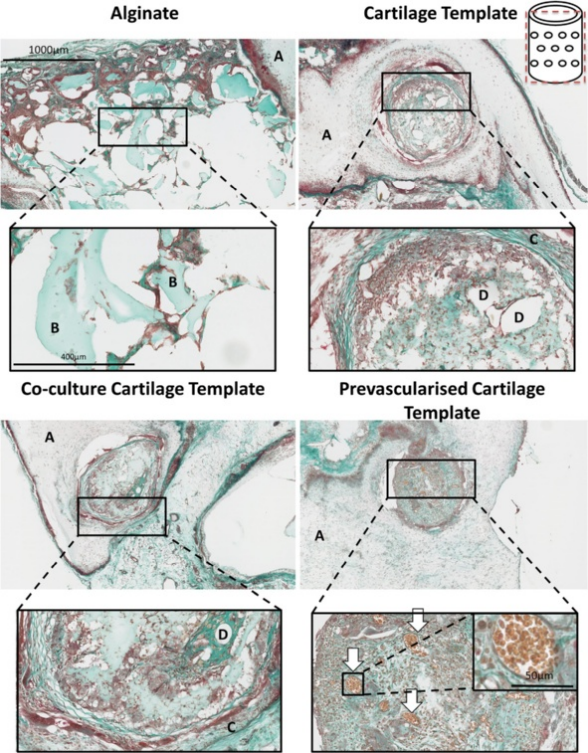
Masson’s Trichrome of the groups after 4 weeks implantation. Images were taken at 5×, 20× and 40×. Schematic of the plane in which the section was taken in in the top right corner. *A* nanofibre mesh, *B* islands of alginate, *C* sGAG rich encapsulation, *D* area of degradation, *arrows* vessels complete with red blood cells

All aggregates were identifiable as a clear circular bundle of cells present within the nanofibre mesh (as indicated by the letter A in Fig. 3) after 4 weeks in vivo. The aggregates are predominately surrounded by alginate and host cells (Fig. 3). As expected there was evidence of degradation in the Cartilage Template and the Co-culture Cartilage Template groups, as indicated by the channels present within the centre of the aggregates (indicated by the letter D in Fig. 3), along with the build-up of fibrous collagen tissue surrounding the aggregate (indicated by the letter C in Fig. 3). However, in the Pre-vascularised Cartilage Template group the degradation was minimal (Fig. 3). There was also positive collagen staining present in all of the aggregates.

### Mineral formation

**Fig. 4:**
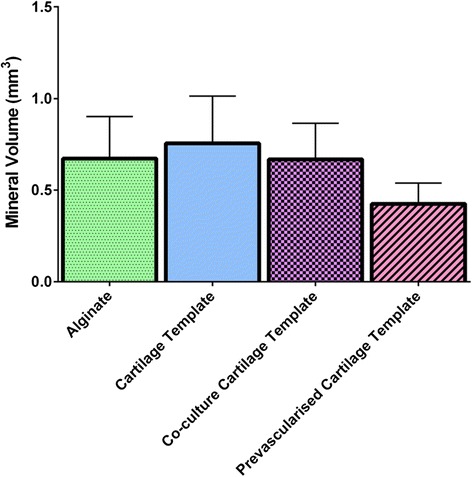
Total mineral volume. Error bars denote standard error (n = 8)

Quantitative mineralisation of the constructs and the surrounding tissue in the hydrogel was analysed from the reconstructed μCT data to determine mineral volume. All groups produced mineral volume between 0.5 and 0.8 mm^3^; however, there was no significant difference between any of the groups after 4 weeks of implantation (Fig. 4).

**Fig. 5:**
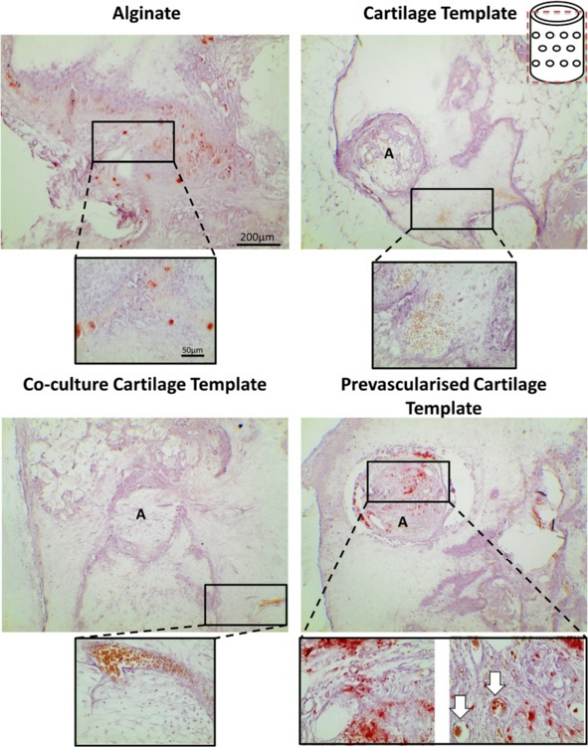
Alizarin Red staining of the groups after 4 weeks implantation. Images were taken at 4× and 40×. Schematic of the plane in which the section was taken in in the top right corner. *Red staining* mineralisation nodules present, *A* aggregates present within the alginate, *arrows* vessels present.

Positive Alizarin Red staining was present in all of the groups; however, the location of the mineral differed by group. The only group to have mineralisation nodules present within the aggregate itself was the Pre-vascularised Cartilage Template group. All of the other groups only had mineralisation nodules present in the surrounding alginate, as seen in Fig. 5. Some of these mineralisation nodules were present in close proximity to mature blood vessels, as indicated in Fig. 5 by the arrows.

### Cell viability

**Fig. 6:**
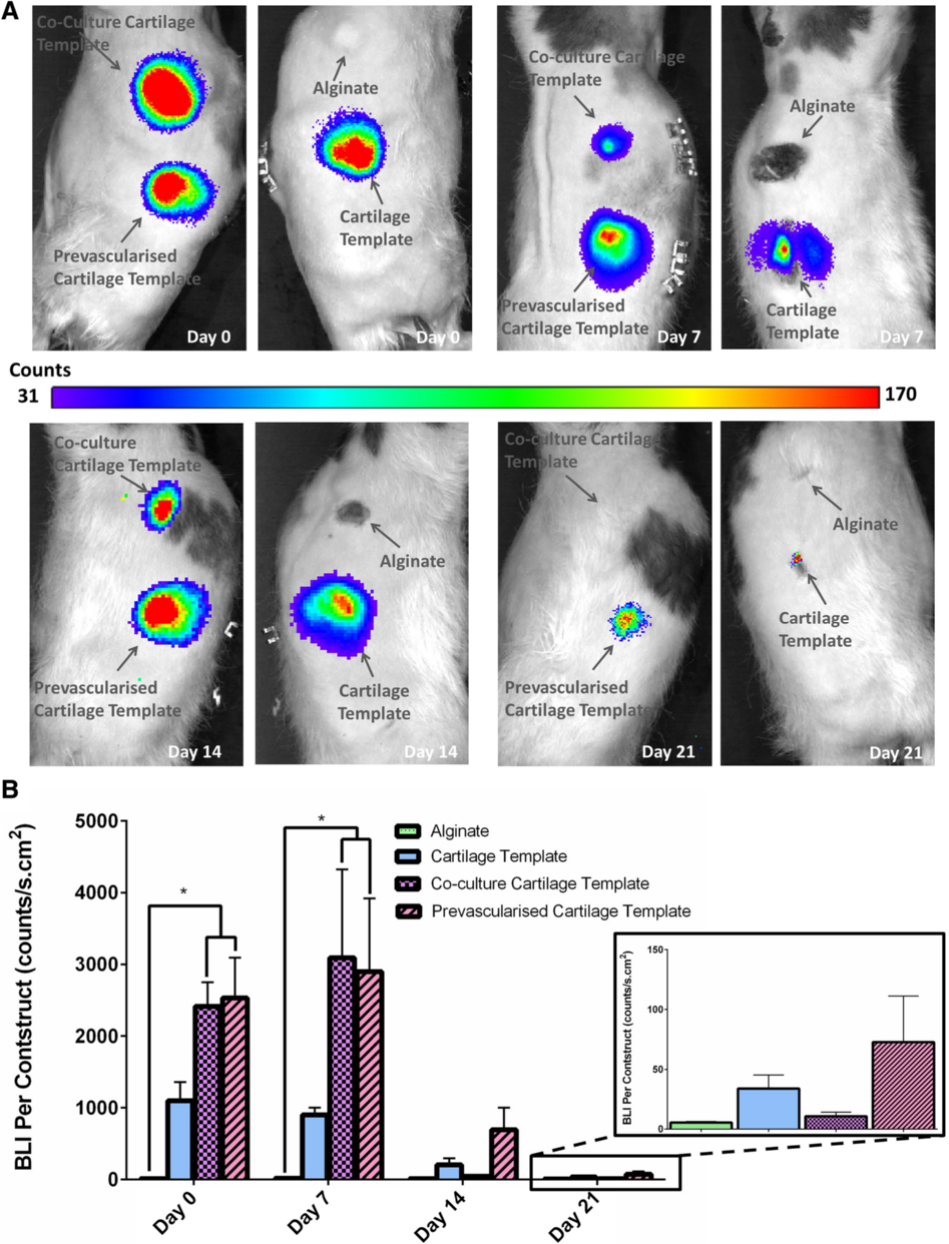
**a** Representative bioluminescent imaging (BLI) heat-maps for representative rat over the time course of the study. **b** Total BLI count of all the groups over the course of the study

BLI data obtained over the course of the study showed that the live cell number from the original cellular aggregate decreased in all groups from the day of surgery to 2 weeks after implantation (see Fig. 6a,b). However, there was a significantly higher BLI signal in the Pre-vascularised Cartilage Template group and Co-culture Cartilage Template group (*p* < 0.05) compared to the Alginate group at both day 0 and day 7. The Pre-vascularised Cartilage Template group also retained more cells compared to the other groups at day 7 (88 % vs. 82–20.5 %) and day 14 (27.4 % vs. 18.3–1.7 %) and by day 21 there was more viable human MSCs present in the Pre-vascularised Cartilage Template group compared to both the Alginate (*p* = 0.1) and Co-culture Cartilage Template groups (*p* = 0.13) (Fig. 6b). BLI imaging was performed on day 28; however, no detectable signal was found.

### Vessel infiltration

**Fig. 7:**
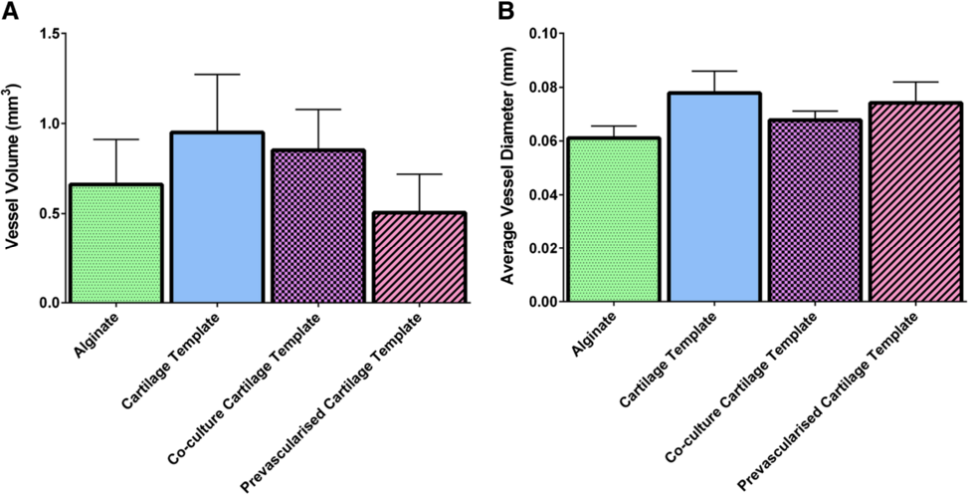
**a** Total vessel volume and **b** average vessel diameter demonstrating the level of vessel formation within the implanted constructs after 4 weeks

**Fig. 8:**
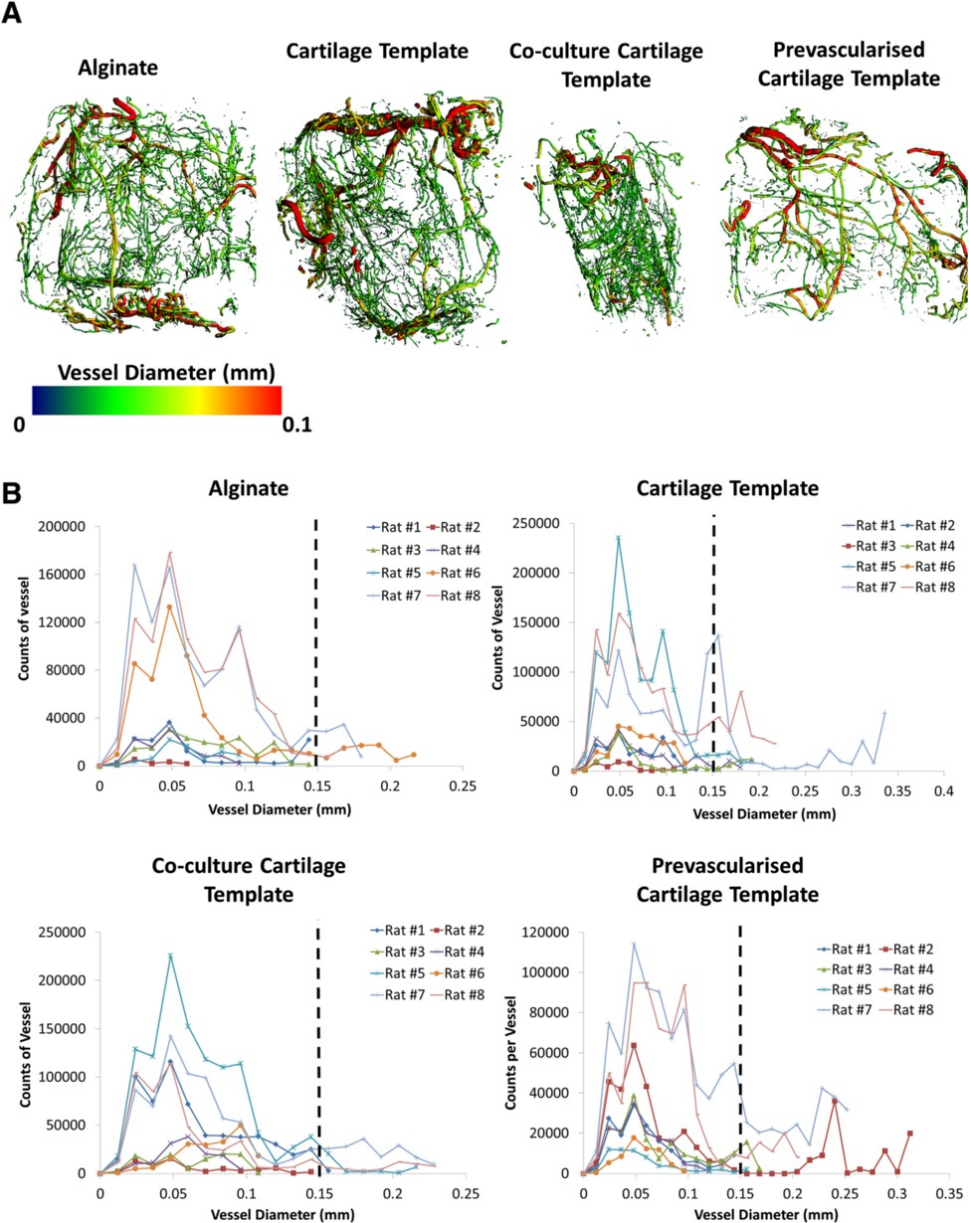
**a** Microcomputed tomography angiography representative images of vessel diameter and **b** histograms of vessel diameters from all planes of the construct for each group, demonstrating the varying vessel thickness of the vessels present after 4 weeks

**Fig. 9:**
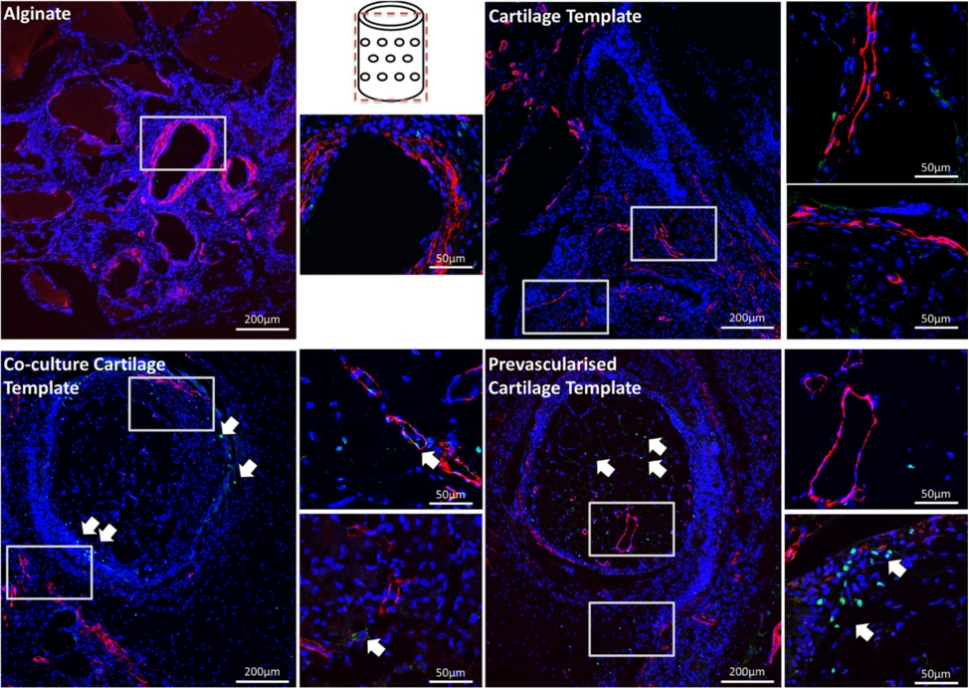
Immunohistochemical staining of the groups after 4 weeks implantation. Boxes denote area of magnification. Images were taken at 10× and 60×. Schematic of the plane in which the section was taken is in the middle of the image. CD31 stained in *green*, nucleus stained in *blue*, smooth actin stained in *red. Arrows* denote presence of CD31 (*green*) within vessel formation

**Fig. 10:**
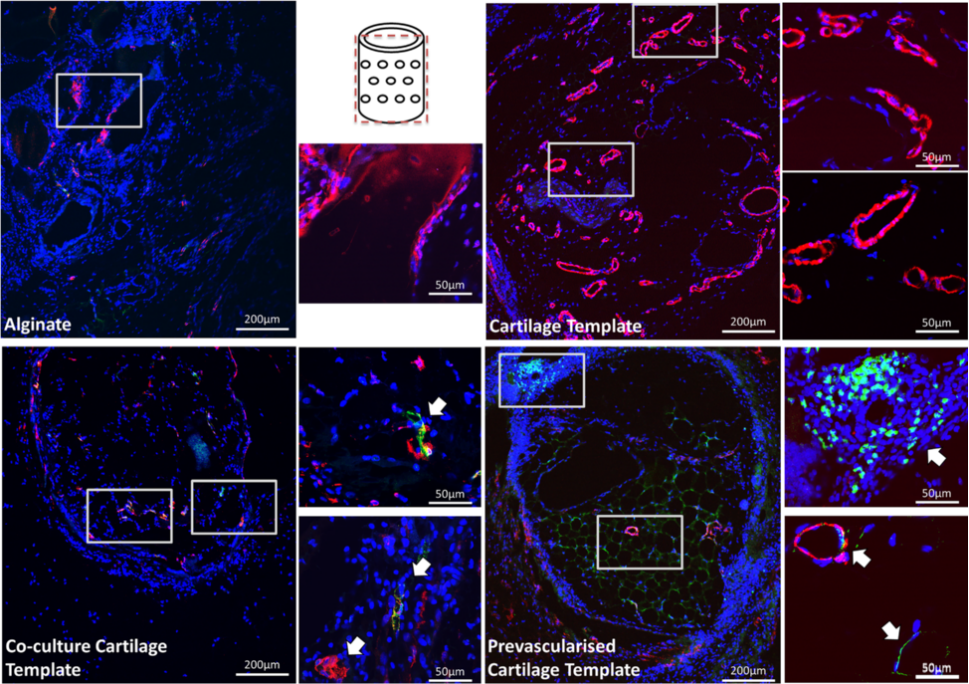
Immunohistochemical staining of the groups after 4 weeks implantation. Boxes denote area of magnification. Images were taken at 10× and 60×. Schematic of the plane in which the section was taken is in the middle of the image. CD146 stained in *green*, nucleus stained in *blue*, smooth actin stained in *red. Arrows* denote presence of CD146 within vessel formation

μCT reconstruction of the explant vasculature illustrated the presence of host blood vessels surrounding the construct and infiltrating the construct through the holes present within the nanofibre mesh (Fig. 7a). Vessel volume was quantified in two ways: 1) total vessel volume; and 2) average vessel diameter. After 4 weeks in vivo there was no significant difference in total vessel volume or average vessel diameter between any of the groups (Fig. 8a,b). To further analyse the data a vessel diameter of 0.15 mm (150 μm) was chosen as a threshold to distinguish between thick and thin vessels and preclude smaller structures (that were unlikely to be mature vessels) from obscuring the results of the analysis. The threshold was chosen on the basis of our immunostaining (described in detail below), which revealed that positively stained α-smooth actin and CD31 and structures with a visible lumen had diameters in the range of 150 μm (see Figs. 9 and 10, described in detail below). Moreover, the majority of vessels in a Sprague–Dawley rat femora are in the range of 120–150 μm [61] and the average vessel diameter achieved within a bone tissue-engineering scaffold implanted in a rabbit alveolar bone defect was 152 μm by 4 weeks [62]. For both the Cartilage Template and the Pre-vascularised Cartilage template groups a large proportion of the vessels were thicker than 0.15 mm. When only vessels with a diameter greater than 0.15 mm are considered, in both the alginate and the Co-culture Cartilage Template group only three out of eight rats had vessels greater than 0.15 mm. These vessels only accounted for 7 % of the overall vessels within the construct. However, in both the Cartilage Template and Pre-vascularised Cartilage Template group five out of eight rats had vessels present with a greater diameter than 0.15 mm and these vessels accounted for up to 14 % of the overall vessels seen within the construct (see Fig. 7b).

Histological staining revealed that there was little vessel formation present within the Cartilage Template and Co-culture Cartilage Template aggregates, but most of the vessels were found outside the aggregates within the alginate (Fig. 3). However, the Pre-vascularised Cartilage Template group was the only group to contain vessels within the aggregate itself complete with red blood cells (Fig. 3, denoted by the arrows).

Immunohistochemical analysis confirmed that mature vessels were present in the surrounding alginate in all of the groups, as indicated by the α-smooth actin staining (Figs. 9 and 10). In contrast the Pre-vascularised Cartilage Template group had mature vessels present within the centre of the aggregates, rather than around the periphery in the surrounding alginate. CD146 and CD31 staining, both endothelial cell markers, revealed that, for both the Co-culture Cartilage Template and the Pre-vascularised Cartilage Template groups, endothelial cells were involved in the formation of these vessels. Moreover, CD31 positive stained cells were present within the structure of some of the vessels (Figs. 9 and 10) indicating that the implanted human endothelial stem cells were involved in the formation of these vessels, as CD31 is only present in human endothelial cells. The staining also showed CD31 positive stained cells were not only present on the periphery of the aggregates but were also present within the surrounding alginate.

## Discussion

This study investigated whether a tissue regeneration approach that incorporates both chondrogenic priming of MSCs to first form a cartilage template, and subsequent pre-vascularisation of the cartilage constructs through the co-culture of HUVECs in vitro, would improve the survival of implanted cells, leading to vessel infiltration and thus mineral formation once implanted subcutaneously in vivo. Our results showed that the pre-vascularised cartilaginous aggregates successfully developed mature vessels (as indicated by α-smooth muscle actin walls and red blood vessels) within the aggregates and retained viable cells from the original aggregate (as indicated by BLI signalling) 21 days after subcutaneous implantation. The pre-vascularised cartilaginous aggregates were also the only aggregates to have mineralisation nodules present within the aggregates. In contrast, chondrogenically primed aggregates, with and without HUVECS, did not have viable cells remaining from the original aggregate after 14 days, had a high vessel volume, but these vessels were not present within the aggregate, and only had mineralisation nodules present in the alginate surrounding the aggregates. Collectively, these results indicate that pre-vascularised cartilaginous aggregates survive for a longer duration following subcutaneous implantation in vivo compared to all other groups, and also that these cellular aggregates contribute to the formation of vessels, with α-smooth muscle actin walls and red blood cells, and increased mineralisation deposition within the construct, which was not observed in aggregates that were not subjected to a combination of chondrogenic priming and pre-vascularisation.

A possible limitation of the study is that MSCs from two male donors were pooled and we did not directly explore whether the human MSCs displayed a donor-dependent response to mineral formation. Previous studies have seen donor variability in the expression of osteogenic growth factors both in vitro [63] and mineral formation in vivo [64]. However, the control groups also contained pooled cells, so the differences observed between the groups cannot be explained by donor variability. A second limitation is that we used MSCs and HUVECs from different donors rather than acquiring both cell types from the same donor. However, it was not feasible to obtain the necessary cell numbers to perform the entire experiment, involving multiple aggregates and priming groups, using cells sourced from the same donors. Future studies could investigate the in vivo potential of one of these groups (i.e. the Pre-vascularised Cartilage Template group) using cells from the same donor source to fully understand the clinical relevance of the approach. Another potential limitation was the length of time the samples were examined for mineral formation (4 weeks). Previous ectopic bone formation models indicate that little to no bone formation will occur until approximately 8 weeks [25, 26, 33, 36–38]. The choice of the 4-week time point allowed us to see both early mineral formation and vessel infiltration and was able to distinguish differences at this early time point. Future studies should investigate the long-term effect of subcutaneous implantation of the pre-vascularised cartilaginous aggregates in order to fully understand their mineralisation potential. Finally, the nanofibre mesh/alginate delivery system with osteogenic growth factors (BMP-2) was used to ensure the retrieval of the aggregates after 4 weeks in vivo, which has proved challenging in other subcutaneous implantation studies [25, 26, 33]. However, as we included an acellular control group, we clearly showed that the results obtained could not be explained by the addition of BMP-2 alone, but that the addition of the cells leads to the differences seen between the groups.

Current bone tissue engineering strategies are limited by challenges arising due to lack of nutrient delivery and waste removal arising from the lack of vasculature [13, 20–26]. Our recent in vitro study reported that a combination of chondrogenic priming and co-culture of human MSCs and HUVECs can lead to the formation of rudimentary vessels and significantly increased the in vitro osteogenic potential of MSC aggregates [54]. Other studies have investigated whether pre-vascularising trabecular bone [43], PCL [44], poly(LLA-co-DXO) [45], collagen GAG [46, 47], and hydroxyapatite [48] scaffolds in vitro would allow faster host integration post-implantation and reported that microvascular networks established in vitro can be maintained when implanted in vivo [43–48]. In this study we investigated whether pre-vascularisation of chondrogenically primed constructs in vitro prior to implantation could overcome limitations of vascularisation and thus degradation of the implanted constructs and uneven mineral distribution. The results from this study show that vessel formation was achieved within the constructs of all groups after 4 weeks implantation (as indicated by μCT angiography, Masson’s Trichrome and α-smooth actin staining). However, the only group to have vessel formation within the aggregates, and not just in the surrounding alginate or in the periphery of the aggregate, was the pre-vascularised cartilaginous aggregates. This may be due to the fact it was also the only group to have rudimentary vessels present prior to implantation (as indicated by CD31 staining). Moreover, it was also the only group to have mature vessels complete with a smooth muscle lining (as indicated by α-smooth actin staining) and red blood cells (as indicated by Masson’s Trichrome). The pre-vascularised cartilaginous aggregates also had the thickest vessel diameters present within the constructs as a whole (as indicated by μCT angiography), with five out of eight of the rats having vessels with diameters between 0.15 and 0.35 mm and these vessels accounted for up to 14 % of the overall vessels present within the constructs. Moreover, the pre-vascularised cartilaginous aggregates were the only group to have viable cells 21 days after implantation. Previous studies have only shown maintenance of viable MSCs to 7 days [57]. Taken together, these results indicate that pre-vascularisation of the cartilaginous aggregates prior to implantation exerts a positive effect on maintenance of the viability of implanted human stem cells in aggregates implanted for 4 weeks in vivo and this is directly associated with the formation of mature vessels present within the centre of the aggregates.

MSCs are a perivascular cell type [65–67], and have been shown to have pro-angiographic effects on endothelial cells when co-cultured in vitro [54, 68]. Our results show both the perivascular role of MSCs in vivo and the pro-angiographic effects on endothelial cells, as the only group to form vessels within the cellular aggregates were the pre-vascularised cartilaginous group, which had both MSCs and HUVECs added to the cartilage template. Interestingly, immunohistochemical staining also revealed that the HUVECs that were added to the already formed cartilage template (in the Co-culture Cartilage Template group and the Pre-vascularised Cartilage Template group) were not just present around the periphery of the aggregates but were also present within the surrounding alginate. Moreover, these HUVECs were shown to play a role in the formation of the mature vessels and integrate with the host cells to form vessels (as indicated by CD31 staining). However, whether it is the human MSCs added during the co-culture or the human MSCs used to form the cartilage template, or the host MSCs that are involved in the formation of these vessels is still unknown. The CD31 stain used was specific for human cells and the persistent staining by 4 weeks after implantation confirms that human cells did persist and may be involved in the formation of the vascular networks. Further studies are needed to elucidate which cells types are involved in forming the vessels observed here.

Unlike other studies [43–48] this study did not use a scaffold. One of the major limitations to current scaffold tissue engineering studies is the inhomogeneous distribution of cells within the construct [69]. This uneven distribution can then lead to heterogeneous properties, fibrous tissue encapsulation [13], and degradation within the centre of the construct, which ultimately leads to the degradation of the scaffold itself [13, 20–26]. Our approach allows the cells to form their own scaffold, mimicking native endochondral ossification, therefore ameliorating the distribution of cells. Previously we have shown that chondrogenically priming MSCs in vitro, to form a cartilage template, provides a suitable scaffold for HUVECs and MSCs to attach, proliferate, infiltrate, and ultimately form rudimentary vessels [54]. This study not only verifies the benefits of this scaffold-less setup but also shows that, even after being implanted for 4 weeks, there was minimal degradation of the centre of the aggregates in the cartilage template of the pre-vascularised cartilaginous aggregates. In contrast, the non-pre-vascularised groups had fibrous tissue present surrounding the aggregates, which can lead to hypertrophy of the cells in the centre of the aggregate and hence the degradation of the centre of the aggregates (seen in the Masson’s Trichrome).

During endochondral ossification, angiogenesis occurs once the cartilage template has formed. This process involves endothelial cells invading through the cartilage canals already present in the developing bone tissue [27–30], and typically occurs between 14 and 18 days of embryogenesis [30, 31]. Therefore in order for mineralisation to occur, the cartilage template must be formed, and vessel infiltration must then occur. Previous studies, which have looked at just the formation of the cartilage template through the subcutaneous implantation of either chondrogenically primed construct [25, 26, 36, 37] or hypertrophic constructs [33, 38], found little to no mineral formation before 8 weeks in vivo. This study found that there was mineralisation present in all of the groups after 4 weeks in this ectopic bone model. Alizarin Red staining of the groups also shows that mineralisation nodules were present predominately in the surrounding Alginate. As the alginate contained BMP-2 this was to be expected. However, the only group that had mineralisation nodules present within the centre of the aggregates was the pre-vascularised cartilaginous aggregates. Our previous in vitro study found that when both MSCs and HUVECs were added to a chondrogenically primed aggregate, mineralisation was reduced, compared to the addition of HUVECs alone [54]. Furthermore, this mineral was characterised by the formation of discrete mineralised nodules rather than homogenous mineralisation throughout the construct, similar to those seen in this study. Researchers have postulated that, in order to mimic bone formation that occurs naturally during the early fetal development, vasculogenesis should be induced prior to osteogenesis in vitro in order to obtain functional bone tissue when implanted in vivo [30, 43, 54]. The results from this study are in agreement with such theories, as the only aggregates to have mineralisation nodules present within the aggregates were also the only group to have mature vessels present within the aggregate. We propose that mineralisation deposition does not occur until after vessel formation, and that this was a possible explanation for why mineralisation was not seen in the chondrogenically primed aggregates (without and with HUVECS alone) as vascularisation within the centre of the aggregate had not occurred. However, mineralisation nodules were beginning to form in the pre-vascularised group but only once mature vessels had formed within the aggregates. Moreover, mineralisation only occurred within close proximately of these vessels. Therefore, it is possible that culturing this group in vivo for longer than 4 weeks will ultimately allow for enhanced mineralisation, but this cannot be verified from the results of the current study and future in vivo investigations are required.

## Conclusions

This study shows for the first time that a tissue regeneration approach that incorporates both chondrogenic priming of MSCs to first form a cartilage template, and subsequent pre-vascularisation of the cartilage constructs through the co-culture of HUVECs and MSCs in vitro improves implanted stem cell viability, vessel formation (as indicated by α-smooth muscle actin walls and red blood vessels) and mineral formation once implanted in vivo. Specifically, the results from this study show that the only group to have mature vessels present within the aggregates after 4 weeks in vivo was the pre-vascularised cartilaginous aggregates. We propose that this vascularisation exerted a positive effect on the viability of implanted stem cells and mineralisation potential of the aggregate, as it was also the only group to have both viable cells 21 days after implantation and mineralisation nodules present within the aggregates. Taken together, these results indicate that endochondral priming of MSC aggregates can increase the survivability of implanted cells, which then contribute to vascularisation of the aggregate and mineral deposition of tissue engineering constructs once implanted in vivo. Future bone tissue engineering strategies could be designed with these conditions in mind such that the factors needed to mimic the endochondral ossification process are incorporated to the point where the constructs themselves can autonomously progress to engraftment, remodelling and ultimately tissue regeneration.

## Abbreviations

ALP: Alkaline phosphatase
ANOVA: Analyses of variance
BLI: Bioluminescent imaging
BMP: Bone morphogenetic protein
BSA: Bovine serum albumin
CP: Chondrogenically primed
DAPI: 4′,6-Diamidino-2-phenylindole
DMEM: Dulbecco’s modified Eagle's medium
EDTA: Ethylenediaminetetraacetic acid
EGM: Endothelial growth medium
FBS: Fetal bovine serum
GAG: Glycosaminoglycan
GFP: Green fluorescent protein
HUVEC: Human umbilical vein endothelial cell
ITS: Insulin–transferrin–selenium
Luc: Firefly luciferase
MOI: Multiplicity of infection
MSC: Mesenchymal stem cell
NBF: Neutral buffered formalin
NGS: Normal goat serum
PBS: Phosphate-buffered saline
PCL: Polycaprolactone
PSL: Penicillin/streptomycin/L-glutamine
ROI: Region of interest
TGF: Transforming growth factor
αMEM: Minimum essential medium alpha
μCT: Microcomputed tomography
